# Correlates of Social Isolation Among Minority Older Adults During the COVID-19 Pandemic

**DOI:** 10.1093/geroni/igab046.1137

**Published:** 2021-12-17

**Authors:** Omolola Adepoju, Daniel Howard, Kendra Smith, Luz Herrera, Daikwon Han, LeChauncy Woodard

**Affiliations:** 1 University of Houston, Houston, Texas, United States; 2 Texas A&M University at College Station, Texas A&M University at College Station, Texas, United States; 3 Smith Research & Consulting LLC, Spring, Texas, United States; 4 Texas A&M University, Fort Worth, Texas, United States; 5 Texas A&M University, Texas A&M University, Texas, United States; 6 University of Houston College of Medicine, Houston, Texas, United States

## Abstract

**Background:**

Over the past year, engagement with older adults has been severely curtailed given the high rates of COVID-19 morbidity and mortality in this population. This study examined the correlates of social isolation among African American and LatinX older adults during the COVID-19 pandemic.

**Methods:**

Working with community-based organizations and senior living centers, we administered a survey to older adults 55+, in the Houston metroplex, between 11/2020 and 01/2021 (n=575). The survey assessed COVID-19 prevention behaviors and health-related social needs. Responses to “How often do you feel lonely or isolated from those around you?” were used to create a dichotomous social isolation dependent variable. The main independent variable, family/community support, was based on responses to the validated question "If for any reason you need help with day-to-day activities such as bathing, preparing meals, shopping, managing finances, etc., do you get the help you need?" Multivariate logistic regression adjusting for socioeconomic status, medical conditions, positive COVID test (for self or family), COVID-19 prevention behaviors, and emergency preparedness levels was used.

**Results:**

Limited family/community support was strongly associated with social isolation (OR=6.2; p<0.01), as was having any chronic condition (OR=2.9, p=0.02). Females and seniors who reported daily social distancing were more likely to report being socially isolated (OR=2.4, p=0.04; OR=1.09; p=0.09, respectively). Of all chronic conditions examined, diabetes was the single strongest predictor of social isolation (OR=2.49, p=0.02).

**Conclusion:**

Being female, having diabetes and limited family/community supports are associated with COVID-19-induced social isolation in African American and Latinx communities.

